# Association between body temperature and leukocyte telomere length in Korean middle-aged and older adults

**DOI:** 10.4178/epih.e2021063

**Published:** 2021-09-08

**Authors:** Carolina García-García, Chol Shin, Inkyung Baik

**Affiliations:** 1 Department of Foods and Nutrition, College of Natural Sciences, Kookmin University, Seoul, Korea; 2 Department of Internal Medicine, Korea University Ansan Hospital, Ansan, Korea

**Keywords:** Body temperature, Telomere shortening, Obesity, Aging, Longitudinal study

## Abstract

**OBJECTIVES:**

Data on associations between body temperature (BT) and leukocyte telomere length (LTL), which has been widely used as a biomarker of cellular senescence in recent epidemiological studies, are limited. Therefore, this study aimed to explore the associations between a normal BT range (35.0-37.5°C) and LTL via 6-year longitudinal observations of 2,004 male and female adults aged 50 or older.

**METHODS:**

BT was obtained by measuring the tympanic temperature, and relative LTL was determined by real-time polymerase chain reaction. Robust regression analysis was used to evaluate the association between the baseline and follow-up LTL values and their differences.

**RESULTS:**

A significant inverse association was found between BT and LTL at baseline. The regression coefficient estimate was -0.03 (95% confidence interval, -0.07 to -0.001; p<0.05). This association was stronger in participants with a body mass index >25 kg/m^2^ and males (p<0.01). However, there were no associations between BT and LTL at follow-up or BT and 6-year longitudinal differences in LTL.

**CONCLUSIONS:**

These findings suggest that having a high BT between 35°C and 37.5°C (95°F and 99°F) may be detrimental for obese individuals in terms of biological aging.

## INTRODUCTION

All living beings are able to experience birth, growth, reproduction, and death in accordance with the life cycle [1]. However, although cells and organisms share the same life cycle stages, their life spans vary. The aging process can be assessed in terms of chronological age (number of years lived) or biological age, which is one’s age relative to the estimated life span as determined by hereditary factors, the state of one’s health, and functioning capacity [2]. Specific aging biomarkers such as genetic indicators, molecular indicators, and environmental factors are used to estimate biological age. It has been estimated that up to 50% of biological variation is caused by genetic factors, of which telomere length has been found to be inversely associated with aging and can thus be used as a potential aging biomarker [3].

Telomeres are hexameric (TTAGGG) tandem repeats found at the end of each eukaryotic chromosome arm that maintain genome stability [4]. Telomere length is considered to be a cellular senescence biomarker similar to a “molecular clock” due to its shortening after each cell’s replication (Hayflick limit) [5]. Leukocyte telomere length (LTL) has been found to be inversely correlated with chronological age and broadly related to other demographic factors, in that LTL can be inherited and is affected by sex [6] and ethnicity [7]. In addition, LTL has been found to be associated with lifestyle factors, chronic diseases, infections, and other stimuli linked to inflammation and oxidative stress [4,8,9], including body temperature (BT) [10].

LTL has been used as a cellular senescence biomarker or a biological clock equivalent in many epidemiological studies [5], mostly due to its dynamic multifactorial relationship to aging-related factors including disease state, environmental factors, and genetics [11], and it has even been found to be associated with parental age at conception [12]. A long LTL measurement has not only been linked to having a good health status (physical fitness, lower inflammation, better redox balance) [13] but also to individuals’ predispositions to unhealthy lifestyles [14,15], diseases [16-18], cancer [19-21], and susceptibility to severe acute respiratory syndrome coronavirus 2 (SARS-CoV-2) infection [22].

Epidemiological studies on the associations between BT and aging or aging biomarkers including LTL are limited. A longitudinal study that analyzed data from over 300 participants to examine the relationship between BT and aging found that a lower BT was associated with a greater survival advantage and that the lower half of the study population according to BT comprised participants with a lower chronological age [23,24]. Some studies found that BT had a positive relationship to body mass index (BMI) and other obesity-related phenotypes associated with shorter LTL [25,26].

The biological mechanisms possibly underlying these associations include increased metabolic rate, insulation of subcutaneous adipose tissue, and the thermogenic effects of hormones produced in adipose tissue [25,26]. However, the effects of obesity on the relationship between BT and LTL have not yet been examined in detail.

In this study, we attempted to evaluate the hypothesis that high values within a normal BT range would be associated with a short LTL, particularly among adults with a high BMI. To explore a possible causal association between a normal BT range and LTL, the present study evaluated the baseline and follow-up LTL values and their differences across a 6-year longitudinal observation period.

## MATERIALS AND METHODS

### Study design and population

This was a longitudinal study embedded in an ongoing prospective cohort study as a part of the Korean Genome and Epidemiology Study. Information on this cohort study is available in previous reports [27,28]. To summarize, the Korean Genome and Epidemiology Study was a population-based cohort study that commenced between 2001 and 2002, which was used as the baseline period, in Ansan, Gyeonggi Province, Korea. The study initially included over 5,000 cohort members and repeated follow-up observations were conducted biannually. Cohort members participated in a questionnaire-based interview, had their anthropometric measurements taken, and attended health examinations. The questionnaire was used to collect the participants’ general characteristics, medical history, and lifestyle habits. In the health examination, biospecimens (whole blood, serum, and urine) were collected [27].

In this study, the blood specimens of 2,314 participants taken during the follow-up period between 2011 and 2012 were used to measure LTL at baseline, and those collected from 2017 and 2018 were used to measure LTL at follow-up. A total of 103 participants (4% of the sample population) did not participate in the measurement of BT and were excluded in addition to those with missing LTL values (n=2) (Supplementary Material 1). Participants with elevated high sensitivity C-reactive protein (hs-CRP; > 3 mg/L) were also excluded due to potential confounding effects of infection in addition to participants who had missing values for anthropometric factors (n=207). Accordingly, a total of 2,002 participants were included in the final analysis. Among these participants, 1,728 (86.3%) provided blood specimens during the follow-up period (2017-2018) and were included in the analysis. There were no significant differences between the baseline LTL values of individuals with and without follow-up LTL values.

### Leukocyte telomere length measurement

Whole blood samples were collected from the study participants and used to analyze LTL utilizing real-time polymerase chain reaction (RT-PCR). In detail, leukocyte genomic DNA was extracted using the QIAamp DNA blood mini kit (Qiagen, Hilden, Germany). After purification and dilution, the samples were quantified using a NanoDrop 1000 spectrophotometer (Thermo Fisher Scientific, Wilmington, DE, USA). The telomere length measurement of single-copy gene 36B4 was conducted using the iQ MultiColor qRT-PCR Detection System (Bio-Rad, Hercules, CA, USA) with telomere primers (5´-GGTTTTTGAGGGTGAGGGTGAGGGTGAGGGTGAGGGT-3´ and 5´-TCCCGACTATCCCTATCCCTATCCCTATCCCTATCCCTA-3´) and 36B4 primers (5´-CAGCAAGTGGGAAGGTGTAATCC-3´ and 5´-CCCATTCTATCATCAACGGGTACAA-3´). For a given DNA sample, the quantification cycle values of the telomere repeat copy number (T) and 36B4 copy number (S) were obtained and used to calculate the T/S ratio for the relative telomere length [29]. The validity of the LTL analysis process was evaluated in a prior study [9].

### Body temperature measurement and anthropometric assessment

BT measurements and anthropometric assessments were conducted by trained personnel who followed body weight a standardized protocol. On the same day that whole blood samples were taken, BT was also measured to the nearest 0.1°C using a digital ear thermometer (Thermoscan; B. Braun Medical Inc., Bethlehem, PA, USA). Body weight (kg) and height (cm) were measured to the nearest 0.1 kg and 0.1 cm, respectively, after the participant removed his or her shoes, and BMI (kg/m^2^) was calculated accordingly. In the present study, obesity was defined as a BMI of 25 kg/m^2^ or greater according to the Asia-Pacific criteria from the World Health Organization guidelines [30].

### Other confounding factors

Information on the participants’ demographic characteristics, smoking status, alcohol consumption habits, and level of physical activity was taken from questionnaire data collected between 2011 and 2012. Physical activity was assessed using a scale composed of five activity intensity categories measured by hours spent in a typical day per intensity level. A total metabolic equivalent (MET/hr) score was calculated by multiplying the hours spent by the MET value (1.0 for sleep or sedentary, 1.5 for very light, 2.4 for light, 5.0 for moderate, 7.5 for vigorous activity) determined based on examples of activities given for each category. Smoking history in terms of pack-years was calculated by multiplying smoking duration in years by the number of cigarette packs smoked per day (1 pack=20 cigarettes). Assessment of hs-CRP concentration and lipid profiles were conducted by a commercial laboratory (Seoul Clinical Laboratories, Seoul, Korea) using serum samples, which were collected after at least 8 hours of fasting. Confounding factors were selected based on previous studies [9,15,16,19,26].

### Statistical analysis

Descriptive statistics were presented as the mean± standard deviation or percentage according to the tertiles of temperature variables. Significant differences among the tertile groups were evaluated using the chi-square test for categorical variables and analysis of variance for continuous variables, and p-values for trends were obtained using the Cochran-Armitage trend test and a generalized linear model procedure. To explore the association between LTL values and their 6-year changes as a dependent variable and the tertile groups for BT, robust regression analysis was used to minimize the effect of outliers of dependent variables. Two models were constructed: an age-adjusted model and a multivariate model adjusted for age (continuous), sex, BMI (continuous), smoking status (non-smokers or smoker), smoking pack-years (continuous), alcohol consumption (non-drinker or drinker), physical activity (total MET-hr/d as quartiles), hs-CRP (continuous), leukocytes count (continuous), menopausal status (yes or no) and presence of chronic diseases such as hypertension, diabetes mellitus, coronary artery disease or cancer (present or absent). Because no evidence of collinearity was found in the statistical test, these covariates were adjusted in the multivariate model. Additionally, further sex-stratified, BMI-stratified (< 25 or ≥ 25 kg/m^2^), and menopause-stratified analyses were conducted. The association analysis between covariates and LTL has been included in Supplementary Material 2. Statistical significance was set at p-value < 0.05 and data were analyzed using SAS version 9.4 (SAS Institute Inc., Cary, NC, USA).

### Ethics statement

All procedures of the study were performed in accordance with the Declaration of Helsinki. The study protocol was approved by the Human Subjects Review Committee at the Korea University Ansan Hospital (IRB approval No. ED0624) and written informed consent was obtained from all participants.

## RESULTS

Among the 2,002 participants (mean age, 58.3 years), 50.6% were female, 11.7% were former or current smokers, 47.5% were former or current alcohol drinkers, 18.5% had been diagnosed with diabetes mellitus or received diabetic treatment, 34.9% had hypertension, and 5.5% had coronary artery disease. Relevant characteristics of the study population are presented in Table 1. Participants were classified into 3 groups according to BT (mean: 36.1°C): 35.0-35.9°C for the first tertile, 36.0-36.2°C for the second tertile, and 36.3-37.5°C for the third tertile. Males were more likely to be in first and second tertiles, where the male-to-female ratio was 1.88 and 1.01, respectively. The mean BMI values tended to increase as BT increased (p<0.01). Most of the participants who consumed alcohol or smoked were in the bottom two tertiles while those with hypertension were more likely to be in the third tertile. The participants in the top tertiles had higher mean levels of total cholesterol and high-density lipoprotein cholesterol.

As a result of the sex-specific analysis, males were found to be more likely to have reduced baseline LTL values as BT increased (p<0.05) than females. In addition, males tended to have a high BMI (p<0.01) as BT increased (Table 2).

Associations between BT and the baseline (n=2,002) and follow-up LTL measurements (n=1,728) are presented in Table 3. A significant inverse association between BT and baseline LTL was observed in the multivariate model. Relative to the first BT tertile, the coefficient estimate (95% confidence interval, CI) for the third tertile was -0.03 (95% CI, -0.07 to -0.01; p<0.05). The BMI-specific analysis results showed a stronger association among participants with a BMI ≥ 25 kg/m^2^ (p<0.01). However, no significant associations with the follow-up LTL values were found. Furthermore, no significant association between BT and 6-year changes in LTL (between the follow-up and baseline values) was found. The findings related to changes in LTL values were not statistically significant.

To explore the associations with the baseline LTL, analyses stratified by sex and BMI were conducted (Table 4). A significant association was observed among males only; relative to the first BT tertile, the coefficient estimate (95% CI) for all male participants was -0.07 (95% CI, -0.12 to -0.02; p<0.01). The BMI-stratified analysis found a similar association only in males with a BMI ≥ 25 kg/m² (p<0.05). However, no significant findings were observed in females. Further analysis stratified by menopause status also resulted in no significant findings. In the tests for interactions, such as BT-sex and BT-BMI, no significant findings were observed (data available upon request).

## DISCUSSION

In the present study, we attempted to test the hypothesis that high values within a normal BT range would be associated with short LTL values and greater attrition of telomeres. We found a significant inverse association between BT and the baseline LTL values of participants, in particular among males with a BMI ≥ 25 kg/m^2^, but did not observe a significant association between BT and follow-up LTL values or 6-year longitudinal changes in LTL.

The rate of living theory states that an organism is more active at a high temperature, and, as a result, organisms will have a higher rate of energy expenditure at high temperatures that in turn shortens their lifespans [31]. Organisms that are efficient at utilizing energy can thus expect greater longevity [32]. One of the pioneering studies of anti-aging effects resulting from lowering an organism’s BT included tests conducted using *Cynolebias* fish and found that, under controlled laboratory conditions, lowering the BT (5°C) could extend their lifespan by nearly 100% [33]. A later study induced mild hypothermia in rodents using repeated injections of tetrahydrocannabinol and marijuana derivate; however, due to the injection’s toxicity, its extreme tranquilizing effect, and rapid tolerance development, it was found to be an impractical agent for inducing chronic mild hypothermia [34]. Other methods, which include biofeedback via meditation, breathing techniques, and a calorie-restricted diet in humans [35,36], have resulted in BT decreases of up to 2°C [37]. Some earlier studies on humans have suggested that there could be an association between a low BT and a longer lifespan [23], which implies that a low BT may be a positive factor for longevity, as prenatal exposure to low environmental temperatures has been found to be linked to longer telomere length, suggesting a strong influence on telomere programming from an early embryonic stage [10]. Another hypothesis states that decreases in BT result in decreases in free radical-mediated damage, which can be determined by comparing the amount and quality of free radical species produced at different temperatures in relation to the activity of endogenous antioxidants such as superoxide dismutase, catalase, and glutathione; hence, the balance of free radicals might be mediated by components affected by temperature [38]. Data have been found to support the hypothesis that aging-associated telomeric changes are accelerated and old cells (in terms of telomeric status) are eliminated after heat exposure [39]; however, to the best of our knowledge, epidemiological data on the relationship between BP and LTL have not yet been found.

Other factors besides temperature, chronological age [2] and obesity [14,40,41] have been found to be associated with short telomeres, as well as sex. Males [16,42] have been found to experience more rapid attrition than females [17]. In the present study, we observed a significant association between higher values within a normal BP range and LTL values among all of the participants. In particular, the association was most pronounced in male participants with a BMI ≥ 25 kg/m^2^, which is defined as obese for Asian adults [30]. It has been found that obesity may be coupled with high BT across different age groups and sex [43] due to the heat loss effect provided by the subcutaneous adipose tissue that acts as an anti-heat-loss barrier, meaning that obese individuals cool less rapidly than non-obese individuals, making them more vulnerable to heat-stress [44]. In conjunction with a high BT, obesity can cause oxidative stress and systematic inflammation [45,46], which may result in a reduced lifespan. Multiple previous studies have confirmed that obesity is linked to shorter telomeres [14,40,41], which we also observed in our findings. However, in terms of telomere biology, it is unclear if lowering the BT of obese individuals within a normal BT range is beneficial. Discrepancies in our findings between male and female participants might be partly due to sex-related physiological differences. It has been suggested that long telomeres in females may be attributed to differences in the endocrine systems of females and males. The female sex hormone, estrogen, may attenuate the attrition rate of telomeres due to its stimulatory effects on telomerase reverse transcriptase gene expression and telomerase activity [47], while estrogen deficiency may be associated with a possible cell senescence response to a withdrawal of telomerase activity [48]. However, LTL attrition appears to be closely related to chronological aging in males but not in females, possibly due to menstruation [49]. A heat-dissipating effect that is enhanced by low BT through vasodilatation has been observed in females. Males, however, are more likely to have a high BT due to their higher resting metabolic rate relative to females, partly due to their higher proportion of muscle mass in the body [50].

The study limitations should be considered when interpreting our findings. We were unable to obtain the LTL values of all of the study participants at the time of the 6-year follow-up. The reduced sample size might lessen the statistical power of the results and lead to a null association between BT and changes in LTL. In addition, residual confounding factors—particularly altered or unobserved confounding factors, such as acute infection—likely affected LTL changes during the follow-up period. Since the study population was limited to Korean adults, the findings cannot be generalized to individuals of other ethnicities. Nevertheless, the findings related to associations may provide biological insights regardless of ethnicity. The strengths of our study include its use of repeated LTL measurements across a large study sample, its consideration of a wide range of confounding factors at the baseline, and its extensive analyses stratified by sex.

In summary, we found an inverse association between relatively high values within a normal BT range (36.3-37.5°C) and low LTL values, especially in male participants with high BMI values (≥ 25 kg/m^2^). However, further studies may be necessary to determine whether losing weight improves telomere length in obese males with a high BT in the normal range.

## Figures and Tables

**Table 1.** Characteristics of the study participants according to the BT tertile

Characteristics		Total	Tertiles of BT [range]			p-value^1^	p-value for trend^2^
			1st tertile [35.0-35.9°C]	2nd tertile [36.0-36.2°C]	3rd tertile [36.3-37.5°C]		
No. of participants, n (%)		2,002 (100)	716 (35.8)	665 (33.2)	621 (31.0)		
Baseline LTL values		1.08±0.41	1.09±0.41	1.08±0.41	1.06±0.40	0.42	0.20
Follow-up LTL values^3^		1.11±0.41	1.10±0.38	1.10±0.42	1.14±0.42	0.11	0.05
Males		49.4	65.4	50.2	30.1	<0.001	<0.001
Age (yr)		58.32±7.34	58.15±7.02	58.12±7.31	58.72±7.66	0.26	0.16
Body mass index (kg/m^2^)		24.64±2.93	24.39±2.69	24.70±2.99	24.86±3.11	<0.05	<0.01
Smokers		11.7	18.0	11.6	4.67	<0.001	<0.001
Smoking (pack-years) among smokers		3.24±10.73	5.11±13.32	2.85±9.55	1.49±7.98	<0.001	<0.001
Alcohol drinkers		47.5	53.8	50.8	36.6	<0.001	<0.001
Presence of hypertension		34.9	31.7	34.6	38.8	<0.05	<0.01
Presence of diabetes mellitus		18.5	16.9	19.6	19.2	0.39	0.27
Presence of coronary artery disease		5.5	6.7	4.2	5.5	0.13	0.29
Presence of cancer		3.8	3.8	3.3	4.5	0.53	0.51
Physical activity (MET-hr/d)^4^		40.76±6.17	41.09±6.63	40.68±5.68	40.46±6.12	0.17	0.07
Biochemical analysis of blood specimens							
	Total cholesterol (mg/dL)	194.50±35.72	190.19±35.59	197.77±36.57	196.97±34.47	<0.001	<0.01
	HDL-cholesterol (mg/dL)	53.88±13.25	53.15±13.45	53.65±12.92	54.95±13.30	<0.05	<0.05
	Triglycerides (mg/dL)	139.33±90.79	137.22±83.96	140.80±94.03	140.19±94.84	0.73	0.55
	High sensitivity C-reactive protein (mg/L)	0.78±0.62	0.82±0.66	0.75±0.59	0.78±0.60	0.16	0.30
	Leukocyte count (10^3^ cells/μL)	5.11±1.46	5.29±1.55	5.02±1.45	5.01±1.34	<0.001	<0.001

Values are presented as the mean±standard deviation or %. BT, body temperature; LTL, leukocyte telomere length; MET, metabolic equivalent; HDL, high-density lipoprotein.

1 Chi-square test for categorical variables and analysis of variance for continuous variables.

2 Obtained using the Cochran-Armitage trend test for categorical variables and a generalized linear model for continuous variables.

3 Analyzed in 1,728 samples.

4 Average daily metabolic equivalent-hours.

**Table 2.** Characteristics according to the BT tertile by sex

Variables		Tertiles of BT [range], °C			p-value for trend^1^
		1st tertile [35.0-35.9]	2nd tertile [36.0-36.2]	3rd tertile [36.3-37.5]	
Sex ratio (male vs. female)		1.88	1.01	0.43	<0.001
Males (n=989)					
	No. of subjects, n (%)	468 (47.3)	334 (33.8)	187 (18.9)	
	Baseline LTL values	1.09±0.41	1.03±0.35	1.01±0.39	<0.05
	Follow-up LTL values^2^	1.08±0.39	1.09±0.44	1.09±0.40	0.62
	Age (yr)	58.54±7.34	57.91±7.20	58.26±7.08	0.66
	Body mass index (kg/m^2^)	24.43±2.60	24.83±2.91	25.04±2.73	<0.01
	Smokers	26.7	22.2	15.0	<0.01
	Smoking (pack-years) among smokers	7.79±15.75	5.63±12.88	4.87±13.97	<0.05
	Alcohol drinkers	69.0	74.6	71.1	0.34
	Presence of chronic diseases^3^	8.1	4.2	7.0	0.27
	Physical activity (MET-hr/d)^4^	41.38±7.32	40.70±5.72	40.51±6.15	0.48
	High sensitivity C-reactive protein (mg/L)	0.82±0.65	0.78±0.58	0.83±0.60	0.76
	Leukocyte count (10^3^ cells/μL)	5.37±1.64	5.08±1.48	5.27±1.42	0.43
Females (n=1,013)					
	No. of subjects, n (%)	248 (24.5)	331 (32.7)	434 (42.8)	
	Baseline LTL values	1.10±0.42	1.11±0.46	1.09±0.40	0.68
	Follow-up LTL values^2^	1.13±0.36	1.11±0.39	1.16±0.42	0.40
	Age (yr)	57.42±6.48	58.33±7.41	58.92±7.90	<0.05
	Body mass index (kg/m^2^)	24.33±2.84	24.56±3.05	24.78±3.26	0.07
	Smokers	1.6	0.9	0.2	<0.05
	Smoking (pack-years) among smokers	0.10±1.08	0.06±0.74	0.03±0.58	0.47
	Alcohol drinkers	25.0	26.9	21.7	0.23
	Presence of chronic diseases^3^	4.0	4.2	4.8	0.60
	Menopause	80.2	82.2	80.4	0.96
	Physical activity (MET-hr/d)^4^	40.54±5.04	40.24±5.04	39.88±5.29	0.11
	High sensitivity C-reactive protein (mg/L)	0.81±0.67	0.72±0.59	0.76±0.61	0.27
	Leukocyte count (10^3^ cells/μL)	5.12±1.37	4.95±1.43	4.91±1.29	<0.05

Values are presented as the mean±standard deviation or %. BT, body temperature; LTL, leukocyte telomere length; MET, metabolic equivalent.

1 Using the Cochran-Armitage trend test for categorical variables and a generalized linear model for continuous variables.

2 Analyzed in 1,728 samples (851 male and 877 female participants).

3 Diabetes mellitus, hypertension, coronary artery disease and/or cancer diagnosis.

4 Average daily metabolic equivalent-hours.

**Table 3.** Associations between BT and the baseline and follow-up LTL values^1^

Models		Tertiles of BT [range], °C		
		1st tertile [35.0-35.9]	2nd tertile [36.0-36.2]	3rd tertile [36.3-37.5]
Baseline LTL values (n=2,002)				
	Age-adjusted model	1.00 (reference)/[716]	-0.01 (-0.05, 0.02)/[665]	-0.03 (-0.06, 0.00)/[621]
	Multivariate model	1.00 (reference)/[716]	-0.01 (-0.04, 0.02)/[665]	-0.03 (-0.07, -0.01)/[621]^*^
	Stratified by BMI (kg/m^2^)			
	<25	1.00 (reference)/[420]	-0.01 (-0.05, 0.04)/[366]	-0.01 (-0.06, 0.04)/[327]
	≥25	1.00 (reference)/[296]	-0.02 (-0.07, 0.03)/[299]	-0.06 (-0.11, -0.01)/[294]^**^
Follow-up LTL values (n=1,728)				
	Age-adjusted model	1.00 (reference)/[576]	-0.02 (-0.06, 0.02)/[576]	0.02 (-0.02, 0.06)/[576]
	Multivariate model	1.00 (reference)/[576]	-0.03 (-0.07, 0.00)/[576]	-0.007 (-0.05, 0.03)/[576]
	Stratified by BMI (kg/m^2^)			
	<25	1.00 (reference)/[364]	-0.02 (-0.07, 0.03)/[318]	-0.004 (-0.06, 0.05)/[269]
	≥25	1.00 (reference)/[267]	-0.05 (-0.10, 0.01)/[261]	-0.007 (-0.06, 0.05)/[249]
Change in LTL values^2^				
	Age-adjusted model	1.00 (reference)/[576]	0.03 (-0.02, 0.09)/[576]	0.03 (-0.02, 0.09)/[576]
	Multivariate model	1.00 (reference)/[576]	0.02 (-0.03, 0.08)/[576]	0.03 (-0.02, 0.09)/[576]
	Stratified by BMI (kg/m^2^)			
	<25	1.00 (reference)/[364]	0.02 (-0.06, 0.09)/[318]	0.02 (-0.06, 0.11)/[269]
	≥25	1.00 (reference)/[267]	0.02 (-0.06, 0.10)/[261]	0.04 (-0.04, 0.13)/[249]

Values are presented as coefficient estimates (95% confidence interval)/[number]. BT, body temperature; LTL, leukocyte telomere length; BMI, body mass index; MET, metabolic equivalent.

1 In the multivariate model, data were adjusted for age, sex, BMI, smoking status (non-smokers or smokers), smoking pack-years, alcohol consumption (non-drinkers or drinkers), physical activity (MET-hr/d as quartiles), serum high-sensitivity C-reactive protein level, leukocyte count, and presence of chronic diseases (diabetes mellitus, hypertension, coronary artery disease and/or cancer diagnosis).

2 Follow-up LTL values minus baseline LTL values.

* p<0.05,

** p<0.01.

**Table 4.** Associations between BT and the baseline LTL values by sex and BMI^1^

Variables		Tertiles of BT [range], °C		
		1st tertile [35.0-35.9]	2nd tertile [36.0-36.2]	3rd tertile [36.3-37.5]
Males				
	All	1.00 (reference)/[468]	-0.02 (-0.06, 0.02)/[334]	-0.07 (-0.12, -0.02)/[187]^**^
	Stratified by BMI (kg/m^2^)			
	<25	1.00 (reference)/[267]	-0.00 (-0.06, 0.05)/[175]	-0.06 (-0.13, 0.01)/[90]
	≥25	1.00 (reference)/[201]	-0.04 (-0.10, 0.02)/[159]	-0.08 (-0.16, -0.01)/[97]^*^
Females				
	All	1.00 (reference)/[248]	0.02 (-0.04, 0.07)/[331]	0.01 (-0.04, 0.06)/[434]
	Stratified by BMI (kg/m^2^)			
	<25	1.00 (reference)/[153]	0.01 (-0.06, 0.08)/[191]	0.02 (-0.05, 0.09)/[237]
	≥25	1.00 (reference)/[95]	0.01 (-0.07, 0.09)/[140]	-0.02 (-0.09, 0.05)/[197]
	Stratified by menopause			
	Yes	1.00 (reference)/[199]	0.02 (-0.03, 0.08)/[272]	0.03 (-0.03, 0.08)/[349]
	No	1.00 (reference)/[49]	-0.02 (-0.15, 0.09)/[59]	-0.08 (-0.20, 0.03)/[85]

Values are presented as coefficient estimates (95% confidence interval)/[number]. BT, body temperature; LTL, leukocyte telomere length; BMI, body mass index; MET, metabolic equivalent.

1 Data were adjusted in a multivariate model for age, sex, BMI, smoking status (non-smokers or smokers), smoking pack-years, alcohol consumption (non-drinkers or drinkers), physical activity (MET-hr/d as quartiles), serum high-sensitivity C-reactive protein level, leukocyte count, presence of chronic diseases (diabetes mellitus, hypertension, coronary artery disease and/or cancer diagnosis), and menopause status (female only).

* p<0.05,

** p<0.01.

## SUPPLEMENTARY MATERIALS

Supplementary Material 1. Study population flowchart.

Supplementary Material 2. Associations between confounding variables and LTL among 2,002 study participants
