# Supplementary material for: Association between body temperature and leukocyte telomere length in Korean middle-aged and older adults
Source: Epidemiol Health. 2021 Sep 8;43:e2021063. doi: 10.4178/epih.e2021063 (PMC8629693; doi:10.4178/epih.e2021063)
Supplement: Supplementary Material 1. — Study population flowchart. [file epih-43-e2021063-suppl1.docx]

**Supplementary Materials**

**Supplementary Material 1.** Study population flowchart.

KoGES: Korean Genome and Epidemiology Study; LTL: Leukocyte telomere length; BT: Body temperature; hs-CRP: high sensitivity C-reactive protein
