# Supplementary material for: Association between body temperature and leukocyte telomere length in Korean middle-aged and older adults
Source: Epidemiol Health. 2021 Sep 8;43:e2021063. doi: 10.4178/epih.e2021063 (PMC8629693; doi:10.4178/epih.e2021063)
Supplement: Supplementary Material 2. — Associations between confounding variables and LTL among 2,002 study participants [file epih-43-e2021063-suppl2.docx]

| **Supplementary Material 2. Associations between confounding variables and LTL among 2,002 study participants** | | | | | | | | | | |
| --- | --- | --- | --- | --- | --- | --- | --- | --- | --- | --- |
| **Variable** | | **Tertiles of Baseline LTL [range]** | | | | | | | ***p*-value**  **for trend*** |  |
|  |  | **1^st^ tertile** | | **2^nd^ tertile** | | | **3^rd^ tertile** | |  |  |
|  |  | **[0.0671-0.8862]** | | **[0.8869- 1.1161]** | | | **[1.1173- 2.9311]** | |  |  |
| Number of participants, (%) | | 667 (33.3) | | 668 (33.4) | | | 667 (33.3) | |  |  |
| Males, % | | 50.2 | | 50.0 | | | 48.0 | | 0.41 |  |
| Age, years | | 59.1±7.6 | | 58.2±7.4 | | | 57.7±6.9 | | <0.001 |  |
| Body temperature, % | | 36.1±0.3 | | 36.1±0.4 | | | 36.0±0.4 | | 0.06 |  |
| Body mass index, kg/m^2^ | | 24.8±2.9 | | 24.7±2.9 | | | 24.5±3.0 | | 0.07 |  |
| Current smokers, % | | 11.7 | | 12.6 | | | 10.9 | | 0.67 |  |
| Smoking pack-years among smokers | | 3.3±10.9 | | 3.3±10.8 | | | 3.1±10.4 | | 0.95 |  |
| Ever alcohol drinkers, % | | 48.0 | | 47.3 | | | 47.1 | | 0.74 |  |
| Presence of hypertension, % | | 31.2 | | 32.9 | | | 34.5 | | 0.30 |  |
| Presence of diabetes mellitus, % | | 19.2 | | 18.4 | | | 17.8 | | 0.53 |  |
| Presence of coronary artery disease, % | | 4.80 | | 5.69 | | | 6.00 | | 0.34 |  |
| Presence of cancer, % | | 4.5 | | 2.8 | | | 4.2 | | 0.25 |  |
| Physical activity, MET-hours/day† | | 40.7±5.9 | | 40.3±6.8 | | | 41.3±5.7 | | 0.09 |  |
| Menopause in women, % | | 39.6 | | 42.0 | | | 43.5 | | 0.34 |  |
| *Biochemical assay in blood* | |  | |  | | |  | |  |  |
|  | Total cholesterol, mg/dL | 195.2±36.8 | | 193.8±35.0 | | | 194.5±35.4 | | 0.70 |  |
|  | HDL-cholesterol, mg/dL | 53.2±13.5 | | 54.1±13.3 | | | 54.4±12.9 | | 0.09 |  |
|  | Triglycerides, mg/dL | 147.9±115.1 | | 134.2±72.3 | | | 135.9±78.5 | | <0.05 |  |
|  | High sensitivity C-reactive protein, mg/L | 0.80±0.63 | | 0.77±0.59 | | | 0.79±0.64 | | 0.72 |  |
|  | Leukocyte count, 10^3^cells/μL | 5.01±1.47 | | 5.11±1.41 | | | 5.22±1.50 | | <0.01 |  |
|  | |  |  | |  |  | |  | | |

Values are presented mean±standard deviation or proportion.
**p*-value for trend was obtained using Cochran-Armitage trend test for categorical and a generalized linear model for continuous variables

LTL, leukocyte telomere length; MET, metabolic equivalent; HDL, high-density lipoprotein
† Average daily metabolic equivalents-hours
